# Hypofractionated radiotheapy using helical tomotherapy for advanced hepatocellular carcinoma with portal vein tumor thrombosis

**DOI:** 10.1186/1748-717X-8-15

**Published:** 2013-01-16

**Authors:** Ji-Yoon Kim, Eun-Jung Yoo, Jeong-Won Jang, Jung-Hyun Kwon, Ki-Jun Kim, Chul-Seung Kay

**Affiliations:** 1 Department of Radiation Oncology, The Catholic University of Korea, College of Medicine, Seoul, South Korea; 2 Department of Internal Medicine, The Catholic University of Korea, College of Medicine, Seoul, South Korea; 3 Department of Diagnostic Radiology, The Catholic University of Korea, College of Medicine, Seoul, South Korea

**Keywords:** Helical tomotherapy, Hepatocellular carcinoma, Portal vein tumor thrombosis

## Abstract

**Background:**

We want to evaluate the efficacy of helical tomotherapy (HT) for treating advanced hepatocellular carcinoma (HCC) with portal vein tumor thrombosis (PVTT).

**Methods:**

We treated 35 patients for unresectable HCC combined with PVTT in whom other treatment modalities were not indicated. The tumor thrombi involved the main trunk of the portal vein in 18 patients (51.4%) and the first or second order branches in 17 patients (48.6%). A median dose of 50 Gy (range: 45–60 Gy) was delivered in 10 fractions. Capecitabine was given concomitantly at a dose of 600 mg/m^2^ twice daily during radiotherapy.

**Results:**

The responses were evaluated via computed tomography. There was a complete response (CR) in 5 patients (14.3%), partial response (PR) in 10 patients (28.6%), stable disease (SD) in 18 patients (51.4%) and progressive disease (PD) in 2 patients (5.7%). The Child-Pugh classification (A vs B) and the Japan integrated staging (JIS) score (2 vs 3) were statistically significant parameters that predicted the response of PVTT (p = 0.010 and p = 0.026, respectively). The median survival, one and two year survival rate of all patients was 12.9 months, 51.4% and 22.2%, respectively. The patients with tumor thrombi in the main portal trunk showed statistically inferior overall survival than patients with tumor thrombi in the portal vein branches (9.8 versus 16.6 months, respectively, p = 0.036). The responders’ median survival was 13.9 months, double 6.9 months as the median survival of the non-responders. No radiation induced liver disease or treatment related mortality was not appeared.

**Conclusions:**

Hypofractionated radiotherapy with HT was effective not only for tumor response but also for survival in the advanced HCC patients with PVTT. And stricter patient selection by Child-Pugh classification and JIS score may maximize the potential benefits of this treatment.

## Background

Hepatocellular carcinoma (HCC) is a common contributor to the cancer incidence and mortality worldwide, and particularly prevalent in East Asia and Africa where hepatitis B and C viral infection is widespread [1]. Locally advanced HCC frequently invades the intrahepatic vasculature and it commonly affects the portal vein. The incidence of portal vein tumor thrombosis (PVTT) is 44–62.8% in all HCC patients according to the autopsy data [2,3] and 31.4-34% according to the clinical data [4,5]. PVTT causes serious problems such as the intrahepatic tumor spread, liver function deterioration and portal vein hypertension and this all leads to intractable ascites, variceal rupture, hepatic encephalopathy and/or death [3].

There are few options to choose for appropriately treating HCC with PVTT. Surgical resection and liver transplantation are limited to a highly selected group of patients who have a good hepatic reserve and a small primary tumor. Transcatheter arterial chemoembolization (TACE) is a widely used treatment for HCC, but it has a lack of efficacy and a high risk of ischemic liver insufficiency when it is performed in patients with PVTT. Various combinations of intraarterial and systemic chemotherapeutic agents have recently been tried in selected patients. However, when performing hepatic intraarterial chemotherapy (HAIC), technical caution is needed to maintain the function of the indwelling catheter and the drug delivery system [6]. So, it is very challenging to treat advanced HCC patients with PVTT. The role of radiotherapy has been gradually expanded from a palliative intent to a curative intent in HCC patients. With the advances in radiotherapy techniques, precise delivery of higher ablative doses is now possible while minimizing the doses to the surrounding normal organs. Therefore, higher tumorcidal dose can be delivered to the target even in the relatively large sized tumor during shorter period than conventional treatment without increase of normal tissue damage.

We have treated HCC patients with PVTT by using helical tomotherapy (HT; Hi-Art system; Accuray, Sunnyvale, CA), and this is capable of intensity modulation as well as imaging guidance. This study reviews our experience using HT plus concurrent capecitabine for HCC patients with PVTT. We evaluated the efficacy and safety of this treatment scheme and analyzed which group of patients might stand to benefit from HT.

## Methods

### Patients

This study included 35 patients diagnosed with at Incheon St. Mary’s Hospital (Incheon, Korea) from February 2006 to April 2010. The diagnosis of HCC was either histologically based (n = 3) or made by the radiographic findings and/or an elevated serum alpha-fetoprotein (AFP) value, with including the presence of risk factors like hepatitis B or C viral infection and underlying liver cirrhosis (n = 32). The presence of PVTT was confirmed by at least two image studies, including contrast enhanced dynamic computed tomography (CT) scans, dynamic enhanced magnetic resonance image or angiograms.

Our inclusion criteria were age > 18 years old, tumor thrombosis in main, 1st or 2nd order branch of portal vein, 3 or less Japanese Integrating Staging (JIS) score, Child-Pugh class A or B, no extrahepatic metastasis and refractory or progressive disease after previous treatment before radiotherapy. We excluded five patients including one patient with Child-Pugh class C and four patients with extrahepatic metastasis from this analysis among the total treated patients (n = 40) because the patients were treated with palliative aim not definitive aim and the number of the patients was too small to analyze. Written informed consent was obtained from all the patients and our Institutional Review Board approved the review of the patients’ data.

### Treatment

For simulation and treatment, the patient was trained to breathe shallowly. When performing simulation with contrast enhanced liver dynamic CT, custom made double vacuum system (BodyFix®, Medical Intelligence, GmbH, Schwabmunchen, Germany) was used for immobilization and abdominal dampening. Two additional series of CT scans during inspiration and expiration were obtained to track the motion of the tumors and other internal organs. The simulation CT images were then transferred to the Pinnacle (v 8.0) planning station (Philips Medical Systems, Andover, MA). The gross tumor volume (GTV) of PVTT with or without intrahepatic tumor was contoured on the portal phase CT. The entire main hepatic tumor was included in the GTV with physician’s decision considering the patient’s liver function, tumor size and the irradiated volume of liver. The internal target volume (ITV) was defined as the summation of the GTVs on the inspiratory and expiratory CT images, and the planning target volume (PTV) was defined with a 1 cm margin around the ITV. The organs at risk (OARs) were the liver, lungs, kidneys, spinal cord, heart, spleen, esophagus, stomach, duodenum and small bowels. Treatment planning was performed using Tomotherapy planning software (Accuray, Sunnyvale, CA) after obtaining the images and contours from the Pinnacle system.

The tumor dose was prescribed to the 95% isodose line encompassing the PTV. For dose constraints for OAR, the dose volumetric parameters were calculated on the basis of the dose-volume histogram (DVH) and they were converted to the equivalent dose in 2 Gy fractions. The mean liver dose and V30 (Vn, the percentage of volume receiving more than n Gy) of the liver were kept less than 28 Gy and 40%, respectively. The volume receiving > 50 Gy was limited to < 1 cc for the stomach and duodenum. The maximal dose was kept below 50 Gy for the spinal cord. The mean dose to each side of kidney was kept below 23 Gy.

Before each treatment, we performed a megavoltage CT (MVCT) scan on the tomotherapy unit. The displacement of tumors and internal organs from their original position on the simulation CT was automatically or manually corrected for three axes (x, y and z) and rotation. However, because MVCT could not show the exact outline of HCC, our landmark of image guidance were bony structure such as spine and rib, outline of liver and porta hepatis. After the corrections, we confirmed that the 95% isodose line encompassed all the tumor volumes on the MVCT and then performed the treatment. Total dose was median 50 Gy (range, 45–60 Gy) in 10 fractions during 2 weeks. The total dose translated to a biologic effective dose (BED) was a median of 75 Gy_10_ (range: 65.3-96 Gy_10_) with the α/β ratio = 10.

HT was delivered once per day, 5 times a week. Chemotherapy with capecitabine (Xeloda; Roche, Nutley, NJ) was administered concomitantly with radiotherapy at a dose of 600 mg/m^2^ twice daily during radiotherapy.

### Follow-up and response evaluation

Physicians evaluated the patients weekly during the treatment, and this included physical examination and the appropriate blood tests. After treatment, patients were evaluated at 2 weeks and 1, 3 and 6 months after radiotherapy and then every 3 months thereafter.

The response of PVTT was evaluated by CT scans and assessing the tumor markers at 1 month after completion of treatment, and then every 2 or 3 months thereafter. The Response Evaluation Criteria in Solid Tumor (RECIST) was used to determine the response of tumor. A complete response (CR) was defined as a complete disappearance of PVTT. Partial response (PR) was defined as at least a 30% decrease of thrombus in the longest diameter. Progressive disease (PD) was defined as at least a 20% increase of PVTT in the longest diameter, and stable disease (SD) was defined as a neither sufficient shrinkage to qualify for PR nor a sufficient increase to qualify for PD. The tumor marker response was also evaluated in patients whom the serum AFP level was elevated above the normal range before tomotherapy. According to the percentage of change compared with the pretreatment level, the AFP response was categorized into a CR (normalization of the AFP level), PR (more than a 50% reduction of the AFP level), PD (more than a 25% increase of the AFP level) and SD (the status met neither PR nor PD).

Treatment related toxicity was evaluated weekly during treatment and at each follow-up visit after treatment. Late toxicity was defined as toxicity occurring 3 months after the completion of treatment. Radiation induced liver disease (RILD) was defined as the development of nonmalignant ascites without disease progression and an anicteric increase in the alkaline phosphatase level of at least two-fold or in the transaminase level of at least five-fold after radiotherapy [7]. The gastrointestinal toxicity and hematologic toxicity were assessed using Radiation Therapy Oncology Group toxicity criteria [8].

### Statistical analysis

To evaluate the association between PVTT response and various parameters, chi-square test, Fisher’s exact test, or *t*-test were used. The patients’ overall survival (OS) was defined as the duration from the start of radiotherapy to the date of death or last follow-up and was calculated from Kaplan-Meier method and the log-rank test was used to compare the effect of each variable on the survival. Statistical significance was set at p-values < 0.05.

## Results

The patient characteristics are shown in Table 1. The median age of the patients was 50 years (range: 40–70 years). The Child-Pugh class was A in 28 patients (80%) and B in 7 patients (20%). Tumor thrombi involved the main trunk of the portal vein in 18 patients (51.4%) and the first or second order branches in 17 patients (48.6%). Thirty patients received median 2 cycles of TACE (range, 1–10 cycles) and one patient underwent percutaneous ethanol injection and high intensity focused ultrasound for the treatment of HCC, but all of them experienced no response or disease progression. For the other 4 patients, HT was performed as the first treatment because other treatment modalities were not indicated. In 24 of 35 patients, median 2 cycles (range, 1–6) of further TACE was performed after HT because they got the restoration of portal vein patency or had multiple HCC to treat.

**Table 1:** Patient characteristics

		**N**	**%**
Age	Median	50 (range, 40 – 70)	
Gender	Male	28	80
	Female	7	20
ECOG	1	18	51.4
	2	17	48.6
Etiology	Hepatitis B virus	27	77.1
	Hepatitis C virus	3	8.6
	Alcoholic	2	5.7
	Other	3	8.6
Child-Pugh classification	A	28	80
	B	7	20
JIS score	2	26	74.3
	3	9	25.7
Pretreatment AFP (ng/ml)	≤ 20	10	28.6
	> 20	25	71.4
Type of HCC	Nodular	20	57.1
	Diffuse infiltrative	15	42.9
Multiplicity	Solitary	24	68.6
	Multiple	11	31.4
Location of thrombi	PV branch	17	48.6
	Main portal trunk	18	51.4
Previous treatment	TACE	30	85.7
	PEI and HIFU	1	2.9
	None	4	11.4

Abbreviations: *ECOG*, Eastern Cooperative Oncology Group; *JIS*, Japan integrated staging; *AFP*, α-fetoprotein; *HCC*, Hepatocellular carcinoma; *PV*, Portal vein; *TACE*, Transarterial chemoembolization; *PEI*, Percutaneous ethanol injection; *HIFU*, High intensity focused ultrasound.

**Figure 1:**
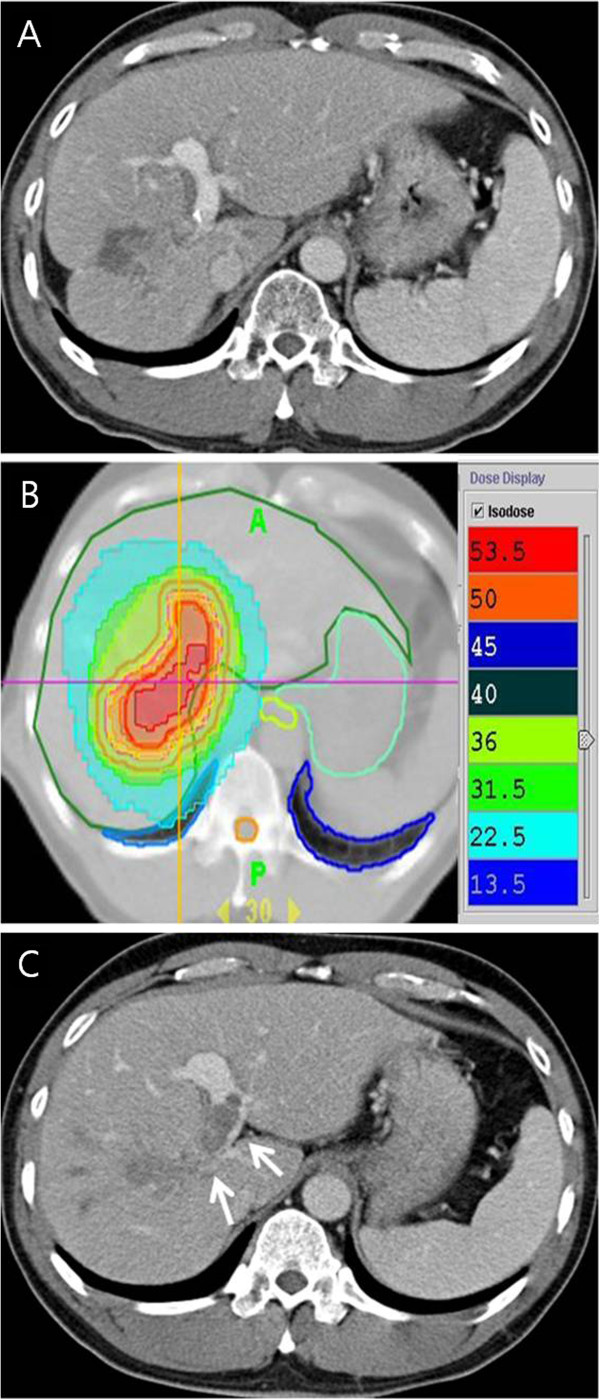
**Case presentation of a patient with PVTT treated with concurrent chemoradiotherapy with HT (50 Gy/10 fractions/2 weeks) and daily administration of capcitabine during radiotherapy.** (**A**) A contrast-enhanced CT in the portal phase shows the diffuse infiltrative HCC with right and main PVTT (white arrows). (**B**) The axial dose distribution of HT with effectively covering the GTV and sparing the adjacent normal organs (**C**) One month after treatment, recanalization of the venous obstruction due to PVTT was achieved.

Median follow up time was 12.9 months (range, 2.9 – 56.6 months). The response of PVTT was evaluable for all the patients. There was a CR in 5 patients (14.3%), PR in 10 patients (28.6%), SD in 18 patients (51.4%) and PD in 2 patients (5.7%). The objective response rate (CR + PR) was 42.9%. Figure 1 shows the case of a patient who achieved a CR of the PVTT. Table 2 showed the relation of the various parameters between responders (CR + PR) and non-responders (SD + PD). Child-Pugh classification and JIS score were statistically significant parameters that predicted the response of PVTT (p = 0.010 and p = 0.026, respectively).

**Table 2:** Factors for predicting the response of portal vein tumor thrombosis

**Factors**		**Responder**	**Non-responder**	**P-value**
		**CR + PR (n = 15)**	**SD + PD (n = 20)**	
Age (yrs)	≤60	7	14	0.163
	>60	8	6	
Gender	Male	12	16	1.000
	Female	3	4	
ECOG	1	8	10	0.845
	2	7	10	
Etiology	Viral	13	17	0.889
	Non-viral	2	3	
Child-Pugh classification	A	15	13	0.010
	B	0	7	
JIS score	2	14	12	0.026
	3	1	8	
Pretreatment AFP (ng/ml)	≤ 20	5	5	0.589
	> 20	10	15	
Type of HCC	Nodular	10	10	0.324
	Diffuse infiltrative	5	10	
Multiplicity	Solitary	8	16	0.093
	Multiple	7	4	
Location of thrombi	PV branch	9	8	0.241
	Main portal trunk	6	12	
BED (Gy_10_, mean ± SD)		70.0 ± 6.5	68.1 ± 11.0	0.530

Abbreviations: *CR*, complete response; *PR*, partial response; *SD*, stable disease; *PD*, progressive disease; *ECOG*, Eastern Cooperative Oncology Group; *JIS*, Japan integrated staging; *AFP*, α-fetoprotein; *HCC*, Hepatocellular carcinoma; *PV*, Portal vein; *BED*, biologically effective dose; *SD*, standard deviation.

P-values are obtained by chi-square test, Fisher’s exact test, or *t*-test.

In 23 patients, intrahepatic tumor was treated with PVTT simultaneously by physician’s decision. The treatment response of intrahepatic tumor was also evaluated. There was PR in 12 patients (52.2%), SD in 8 patients and PD in 3 patients.

Of the 25 patients who had elevated AFP levels before radiotherapy, the response of the AFP was evaluable in 24 patients. Seventeen patients (70.8%) achieved PR according to the AFP level. Three patients (12.5%) showed SD. Four patients (16.7%) had progression of the serum AFP level.

**Figure 2:**
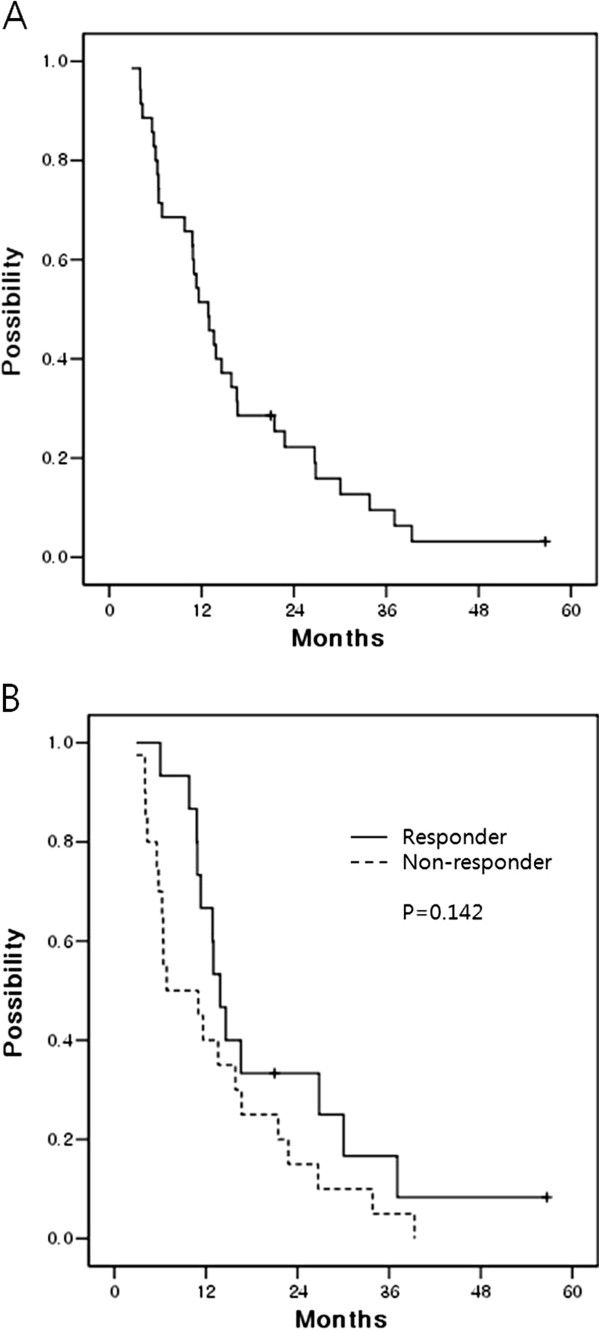
**(A) The median overall survival of all the patients was 12.9 months.** One-year OS rate was 51.4 ± 8.5% and 2-year OS rate was 22.2 ± 7.1%, respectively. (**B**) The overall survival rates according to the response of PVTT. For the responders (CR + PR), the median survival was 13.9 ± 1.1 months, double 6.9 ± 5.1 months as the median survival period of the non-responders (SD + PD). One-year survival rate of the responders and non-responders was 66.7 ± 12.2% and 40.0 ± 11.0%, respectively. Two-year survival rate of responders and non-responders was 33.3 ± 12.2% and 15.0 ± 8.0%, respectively.

At the time of analysis, 33 patients had died and 2 patients were alive. The OS duration for all the patients was a median of 12.9 months (range: 2.9 – 56.6 months). The OS curve for all the patients is presented in Figure 2A. One-year OS rate was 51.4 ± 8.5% and 2-year OS rate was 22.2 ± 7.1%, respectively.

For the treatment responders, the median survival duration was 13.9 ± 1.1 months, double 6.9 ± 5.1 months as the median survival duration of the non-responder, yet statically significant difference was not noted (p = 0.142) (Figure 2B).

On the log-rank test, the involvement of the main portal trunk was a significant unfavorable prognostic factor for OS. Patients with PVTT which involved main portal trunk survived 9.8 ± 4.2 months and patients whose PVTT involved first or second order portal vein branches survived 16.6 ± 5.9 months (p = 0.036). Table 3 summarized the univariate analyses for the OS.

**Table 3:** Factors for predicting the overall survival

**Factors**		**Median survival**	**P-value**
		**(months)**	
Age (yrs)	≤60	9.8 ± 4.9	0.585
	>60	13.6 ± 2.1	
Gender	Male	12.9 ± 1.9	0.293
	Female	11.6 ± 1.0	
ECOG	1	15.9 ± 4.0	0.070
	2	9.8 ± 3.1	
Etiology	Viral	11.3 ± 1.4	0.344
	Non-viral	13.6 ± 2.2	
Child-Pugh	A	13.0 ± 1.5	0.309
Classification	B	6.4 ± 0.8	
JIS score	2	13.0 ± 1.4	0.235
	3	5.8 ± 0.3	
Pretreatment	≤ 20	15.9 ± 1.6	0.161
AFP (ng/ml)	> 20	10.9 ± 1.0	
Type of HCC	Nodular	11.3 ± 0.7	0.853
	Diffuse infiltrative	13.6 ± 4.4	
Multiplicity	Solitary	11.3 ± 1.3	0.534
	Multiple	13.9 ± 2.1	
Location of thrombi	PV branch	16.6 ± 5.9	0.036
	Main portal trunk	9.8 ± 4.2	
BED (Gy_10_)	< 75	13.0 ± 1.4	0.901
	≥ 75	9.8 ± 4.7	
PVTT response	CR + PR	13.9 ± 1.1	0.142
	SD + PD	6.9 ± 5.1	

Abbreviations: *ECOG*, Eastern Cooperative Oncology Group; *JIS*, Japan integrated staging; *AFP*, α-fetoprotein; *HCC*, Hepatocellular carcinoma; *PV*, Portal vein; *BED*, biologically effective dose; *CR*, complete response; *PR*, partial response; *SD*, stable disease; *PD*, progressive disease.

P-values are obtained by log-rank test.

The treatment-related toxicities are presented in Table 4. The acute hematologic or gastrointestinal toxicities were transient. In terms of liver complications, no apparent RILD was observed according to the previously defined criteria. From one to 3 months after the completion of radiotherapy, analysis of the liver function using the Child-Pugh classification showed no change in class in 23 patients. Twelve patients experienced the Child-Pugh classification deterioration with 10 patients deteriorating from A to B and 2 patients deteriorating from B to C. Among them, 2 patients experienced local tumor progression and 2 patients had progression of distant metastasis. No Hand-foot syndrome related to capecitabine appeared.

**Table 4:** Treatment related acute and chronic toxicity according to Radiation Therapy Oncology Group (RTOG) toxicity criteria

		**Grade**	**1**	**2**	**3**	**4**
Acute	Hematologic	Hemoglobin	0	2	0	0
		Leukocytes	4	5	2	0
		Platelets	4	7	1	0
	Gastrointestinal		10	3	0	0
Chronic	Gastrointestinal		0	0	1	1

Late gastrointestinal toxicity appeared in 2 patients. One patient experienced a duodenal ulcer bleeding and this corresponded to grade 3 gastrointestinal toxicity at 5 months after radiotherapy. He received blood transfusion and endoscopic argon ablation therapy. The other patient was diagnosed with duodenal ulcer at 7 month after radiotherapy and managed with proton pump inhibitor. But after 1 month, a small perforation developed in the duodenal ulcer area and this resulted in grade 4 gastrointestinal toxicity. The site of the perforation was successfully sealed with histoacryl and lipiodol by endoscopic injection.

## Discussion

Considering that almost HCC patients with PVTT have a poor hepatic reserve associated with decreased portal blood flow and underlying liver cirrhosis, preservation or restoration of liver function is as important for their prognosis as the achievement of tumor control. With the advances of 3-dimensional conformal radiotherapy (3D-CRT), it is possible to minimize the irradiation of the normal liver with increasing the tumor dose. Promising results have been reported by dose escalation studies for small HCCs [9]. Similarly, in HCC patients with accompanying PVTT, the response rate (CR + PR) was improved to 39–45.8% with increasing the total dose in recent retrospective series of 3D-CRT. BED over 58 Gy_10_ was a significant predictor for tumor response and overall survival [10,11]. So, we have tried to prescribe the dose as high as possible because dose response relationship was noted for HCCs with or without PVTT [9-11].

With the advent of intensity modulated radiotherapy (IMRT), capable of generating complex spatial dose distributions, high dose of radiation can be more safely focused to the target. HT has facilitated dedicated IMRT delivery because of the continuously rotating 6-MV linear accelerator with a dynamically positioned multileaf collimator every 7 degrees around the patient. A further advantage of the tomotherapy unit is to minimize the set up errors by the MVCT guidance. With HT, treating an entire huge HCC or simultaneous irradiation of multiple targets can be safely performed with sparing the normal OARs [12,13].

There is currently little published data on treating HCC patients who have PVTT with the recent advanced radiation techniques. McIntosh et al. reported on the treatment outcomes with HT plus concurrent capecitabine for 20 unresectable HCC patients. The HCC was accompanied by PVTT in 8 of the patients. The total dose was 50 Gy in 20 fractions. A PR or SD by the RECIST was achieved in 15 of the 16 evaluable patients. The response of 8 patients who have PVTT was not evaluated separately [14]. Choi et al. performed stereotactic body radiation therapy using Cyberknife in 9 HCC patients whose target volume was only PVTT. The total dose was 30–36 Gy in 3 fractions. The response rate of the PVTT was CR in 1 patient (11.1%) and PR in 3 patients (33.3%) by the WHO criteria, but the sample size was too small [15]. In the present study, the objective response of the PVTT was CR in 5 patients (14.3%), PR in 10 patients (28.6%), SD in 18 patients (51.4%) by the RECIST criteria. Unfortunately, precise comparison between recently published studies and this study was impossible because of different criteria of patient selection and dose schedule of radiotherapy. However, although this study included a larger proportion of patients with poor prognostic factors including Child-Pugh class B and ECOG scale 2, it showed promising results in the response rate and overall survival similar or superior to previously published studies in which the authors reported median overall survival time from 9 to 10 months [10,11]. Table 5 summarized the treatment outcomes of studies which used radiotherapy for HCC patients with PVTT. We prescribed high tumorcidal dose using hypofractionated schedule with a large fraction size during short course of radiotherapy. Moreover, it may be valuable for HCC patients with PVTT when considering their limited life expectancy. After careful investigating our results, we found that the patients’ response rates were similar between the patients with ECOG scale one and two (8/18 vs. 7/17) and the responders tended to survive longer than non-responders. In addition, the median survival time even in the patients with ECOG scale 2 was 9.8 months and this was similar as the patients with good performance status treated with 3DCRT in the other studies [10,11]. In general, it takes 5 to 6 weeks to treat the patients with HCC accompanying PVTT using 3DCRT, on the other hand, our treatment duration using hypofractionated radiotherapy with HT was only 2 weeks and this treatment could be easily performed even in the patients with ECOG scale 2. Thus, we may guess that even the patients with ECOG scale 2 if they achieved good response after treatment can survive longer than we supposed. However, because there was no statistical significance, proceeding trials should be required to make it certain.

**Table 5:** Comparison of the literatures for radiotherapy in hepatocellular carcinoma patients with portal vein tumor thrombosis

***Reference***	***N***	***Techniques***	***Fraction size (Gy)***	***Total dose (Gy)***	***Response (%)***	***Median survival (months)***
Toya [10]	38	3D-CRT	1.8–4	40 (range, 17.5–50.4)	CR = 15.8	9.6
					PR = 28.9	
Kim [11]	59	3D-CRT	2–3	30–54	CR = 6.8	Responder: 10.7
					PR = 39.0	Non-responder: 5.3
McIntosh [14]	20	Helical Tomotherapy	2.5	50	PR = 6.2	9.6
					SD = 87.5	
Choi [15]	9	Cyberknife	10–12	30–36	CR = 11.1	8
					PR = 33.3	
Current study	35	Helical Tomotherapy	4.5–6	50 (range, 45–60)	CR = 14.3	12.9
					PR = 28.6	Responder 13.9
						Non-responder 6.9

Abbreviations: *Gy*, Gray; *PVTT*, Portal vein tumor thrombosis; *3D-CRT*, 3 dimensional conformal radiotherapy; *CR*, Complete response; *PR*, partial response; *SD*, Stable disease.

A combination scheme of radiotherapy and systemic therapy might be beneficial for locally advanced HCC patients because they often present with multiple intrahepatic or disseminated extrahepatic diseases even at the initial presentation, or they quickly experience a growth of the intra or extrahepatic metastasis out of the radiation field after radiotherapy. Improved treatment outcomes with a combination of radiotherapy and systemic therapy were reported by a series of 3D-CRT. In the pilot study of localized chemoradiation therapy from South Korea, Han et al. reported a superior outcome in advanced HCC with PVTT. They treated the patients with 3D-CRT and HAIC with 5-fluorouracil for Child-Pugh class A patients. The median survival time and 3 year survival rate were 13.1 months and 24.1%, respectively. The response rate after treatment was 45% and the responders demonstrated better survival than the non-responders (median survival, 19.9 months vs. 11.4 months, respectively, p = 0.033) [6]. The treatment result of their study was superior long term survival to our study. However, they included only patients with Child-Pugh class A and the proportion of patients with main portal trunk invasion was limited to 32.5% [6]. On the other hand, our study included the patients with Child-Pugh class B (20%) and the proportion of main portal trunk invasion was 51.4%. As a concurrent chemotherapeutic regimen, the present study used capecitabine, which has both a systemic effects and radiosensitization. The patients well tolerated the concurrent chemoradiation and the chemotherapy-related acute toxicities were mild and transient.

As for staging, we choose the JIS system that integrates both liver function and tumor-node-metastasis staging by the Liver Cancer Study Group of Japan. The discriminatory value of JIS system is more noted among the intermediate- and advanced-stage HCC patients when compared with other staging systems such as Okuda classification or CLIP scoring for HCCs [16]. As most HCC patients with PVTT have far advanced staging scores, JIS system might identify the subgroup of patients who could be benefited from short course high dose radiotherapy that was noted in this study. Actually, we found only one responder after hypofractionated radiotherapy using HT among the patients with JIS score 3.

Through the DVH analysis of the 3D-CRT series, the hepatic toxicity following radiotherapy could be correlated with several dosimetric parameters. The mean liver dose, the V30 or the V50% (Vn%: the percentage of the liver volume receiving more than n% of the isocenter dose) and the normal tissue complication probability (NTCP) are widely accepted to be predictive of RILD [17,18]. While delivering a tumorcidal dose for tumor control, the advanced RT techniques is expected to decrease the incidence of RILD. Cheng et al. compared the differences of the dose-volume data between 3D-CRT and IMRT for HCC patients. They found that IMRT could significantly reduce the NTCP (23.7% vs. 36.6%, respectively, p = 0.009), but significantly increased the mean liver dose (29.24 Gy vs. 25.04 Gy, respectively, p = 0.009) [19]. Lee et al. analyzed the dosimetric parameters of 3D-CRT, linac-based IMRT and HT. HT could achieve the best tumor coverage, but HT’s mean liver dose was highest among the techniques [20]. Although liver toxicity is frequently combined with the influence of chemotherapy, disease progression or hepatitis B viral activation, further study is needed to determine if HT can reduce the incidence of RILD while improving the dose conformity with the price of an increased mean hepatic dose.

Regarding the gastrointestinal complication following hypofractionated radiotherapy in treating PVTT, gastric or duodenal ulcer could be a serious toxicity due to the proximity of portal vein to the stomach or duodenum. We experienced 2 patients suffered from duodenal ulcer bleeding (5.7%) which was managed with medication or endoscopic procedures. Other study using HT for 20 patients reported 1 patient with melena secondary to gastric ulcer bleeding. Considering that their total dose was 50 Gy in 20 fractions which was lower compared with current study of 45–60 Gy in 10 fractions, hypofractionated radiotherapy with HT in the present study was performed safely [14].

## Conclusions

This is the first study to treat HCC patients with PVTT by hypofractionated radiotherapy schedule during short period using HT. Radiation dose escalation was safely performed and treatment response rate and overall survival was good as expected with high dose of radiation even in the patients with poor performance status (ECOG scale 2). Strict patient selection through Child-Pugh class and/or JIS score will maximize potential benefits of our treatment. However, hypofractionated radiotherapy using HT (or IMRT) in advanced HCC with PVTT needs to be further investigated through prospective clinical trial in large number of HCC patients.

## Abbreviations

HCC: Hepatocellular carcinoma; PVTT: Portal vein tumor thrombosis; TACE: Transcatheter arterial chemoembolization; HAIC: Hepatic intraarterial chemotherapy; HT: Helical tomotherapy; AFP: Alpha-fetoprotein; CT: Computed tomography; GTV: Gross tumor volume; ITV: Internal target volume; PTV: Planning target volume; OARs: Organs at risk; MVCT: Megavoltage CT; BED: Biologic effective dose; RECIST: Response Evaluation Criteria in Solid Tumor; CR: Complete response; PR: Partial response; PD: Progressive disease; SD: Stable disease; RILD: Radiation induced liver disease; OS: Overall survival; DVH: Dose-volume histogram; Vn: The percentage of volume receiving more than n Gy; ECOG: Eastern Cooperative Oncology Group; JIS: Japan integrated staging; HR: Hazard ratio; CI: Confidence interval; IMRT: 3-dimensional conformal radiotherapy (3D-CRT); IMRT: Intensity modulated radiotherapy; Vn%: The percentage of the liver volume receiving more than n% of the isocenter dose; NTCP: Normal tissue complication probability.

## Competing interests

The authors declare that they have no competing interests.

## Authors’ contribution

CSK planned the fractionation schedule of this study. CSK, JWJ and JHK performed the management and evaluation the patients. KJK interpreted the radiological findings. JYK participated in the acquisition of data and drafted the manuscript. CSK performed the analysis and interpretation of data and edited the draft. All the authors read, participated in the edition of the manuscript and approved the final manuscript.
